# Comparison of the TOFscan and the TOF-Watch SX during pediatric neuromuscular function recovery: a prospective observational study

**DOI:** 10.1186/s13741-021-00215-2

**Published:** 2021-12-10

**Authors:** Hyung-Been Yhim, Young-Eun Jang, Ji-Hyun Lee, Eun-Hee Kim, Jin-Tae Kim, Hee-Soo Kim

**Affiliations:** 1 Department of Anesthesiology and Pain Medicine, Seoul National University Hospital, Seoul, Republic of Korea; 2 Department of Anesthesiology and Pain Medicine, College of Medicine, Seoul National University, #101 Daehak-ro, Jongno-gu, Seoul, 03080 Republic of Korea

**Keywords:** TOFscan, TOF-Watch SX, Acceleromyography, Pediatric

## Abstract

**Background:**

TOFscan is a three-dimensional acceleromyography neuromuscular monitoring device that does not require initial calibration before muscle relaxant injection. This study aimed to compare TOFscan with TOF-Watch SX, the currently widely accepted uni-dimensional acceleromyography, for use among the pediatric population. We aimed to assess the agreement between TOFscan with TOF-Watch SX in the pediatric population’s neuromuscular recovery.

**Methods:**

A total of 35 children aged 6–12 years were enrolled. Prior to any muscle relaxant injection, TOFscan and TOF-Watch SX were applied at each opposite arm and monitoring began concurrently throughout neuromuscular recovery. Calibration was performed for TOF-Watch SX, and train-of-four values were recorded every 15 s. Agreement between the two devices was evaluated with Modified Bland-Altman analysis.

**Results:**

The bias between TOF-Watch SX and TOFscan were all within the 95% limits of agreement. The bias and standard deviation were smaller and the limit of agreement was narrower in the normalized group than in the non-normalized group [normalized bias −0.002 (95% CI, −0.013 to 0.010), standard deviation (SD) 0.111 vs non-normalized bias 0.010 (95% CI, −0.003 to 0.0236), SD 0.127].

**Conclusions:**

TOFscan reliably demonstrated lack of bias and good concordance with TOF-Watch SX throughout the neuromuscular recovery, especially when normalized. Despite technical limitations, the two devices were unbiased along the path of spontaneous and pharmacological reversal in pediatric patients.

**Trial registration:**

ClinicalTrials.gov NCT03775603. Registered on 13 March 2018

**Supplementary Information:**

The online version contains supplementary material available at 10.1186/s13741-021-00215-2.

## Introduction

When using neuromuscular blocking agents (NMBA), quantitative neuromuscular monitoring is mandatory to optimize intubation time, monitor intraoperative muscle relaxation, determine adequate pharmacologic reversal agents, and reduce postoperative residual paralysis (Murphy et al., 2013; Naguib et al., 2017; Brull & Kopman, 2017; Fortier et al., 2015; Naguib et al., 2018). National guidelines, such as World Federation of Societies of Anaesthesiologists (WFSA) International Standards for Safe Practice of Anesthesia, and French Society of Anesthesia and Intensive Care (SFAR), recommend incorporating objective neuromuscular monitoring into daily practice (Plaud et al., 2020; Gelb et al., 2018; Nemes & Renew, 2020). Although many anesthesiologists rely on subjective evaluation, dependence on such clinical parameters has limited success (Naguib et al., 2018).

Clinical assessment of adequate reversal is challenging in children given difficulties with communication and non-compliance with instructions. Previous evidence indicates that 10–28% of children experience postoperative residual block (i.e., train-of-four (TOF) ratio <0.9), with 6.5% displaying severe block (i.e., TOF ratio <0.7) (Ledowski et al., 2015; de Souza et al., 2011). Complications due to residual block can be detrimental given that children have smaller oxygen reserves and are more vulnerable to airway collapse (Fortier et al., 2015; von Ungern-Sternberg et al., 2006; Hardman & Wills, 2006).

The current standard device for determining the depth of muscle relaxation is the TOF-Watch SX (Organon, Swords Co., Dublin, Ireland), which is a one-dimensional acceleromyograph requiring initial calibration (Colegrave et al., 2016). Without calibration, TOF-Watch SX transmits erroneous information regarding the degree of residual neuromuscular blockade (Martin-Flores et al., 2012). Acceleromyography-based devices frequently overestimate TOF ratio more than mechanomyography or electromyography (Suzuki et al., 2006). Therefore, when using acceleromyography, “normalization” of taking baseline TOF ratio into account by dividing the expected TOF ratio with baseline TOF ratio at each interval throughout the neuromuscular recovery is recommended (Claudius et al., 2009).

Recently, TOFscan (Drager Technologies, Canada), a new device also using accelerometry, has emerged on the market. TOFscan does not require onerous calibration and differs from the TOF-Watch as it measures three-dimensional acceleration. A previous evaluation of the agreement between the two devices during neuromuscular recovery in adults indicated good agreement when TOF-Watch SX was calibrated and normalized (Murphy et al., 2018).

This study aimed to compare the performance of TOF-Watch SX and TOFscan in children. We hypothesized that TOFscan measures would be comparable to TOF-Watch SX’s among children aged ≤12 years.

## Materials and methods

### Patient recruitment

A prospective, observational clinical trial was conducted between December 2018 and August 2019 at a single tertiary medical center. The study was approved by the SNUH Institutional Review Board (1811-137-989) and was registered at ClinicalTrials.gov (https://clinicaltrials.gov/ct2/show/NCT03074968). Each participant and their parents were provided a verbal explanation of the study and given the opportunity to ask questions. Written informed consent was obtained from participants aged ≥7 years and one of their parents. Verbal consent was obtained from participants aged <7 years, in addition to written informed consent from one of their parents. All procedures were conducted in compliance with the principles of the Helsinki Declaration.

In total, 39 children aged ≤12 years were screened, of whom 35 were enrolled. All children were classified as American Society of Anesthesiologists physical status I–II and scheduled for elective surgery under general anesthesia. Exclusion criteria were as follows: body mass index ≥30 kg∙m^−2^; presence of neuromuscular disease, myopathy, susceptibility to malignant hyperthermia, renal insufficiency (i.e., estimated glomerular filtration rate ≤60 ml/min), or liver disease; surgery with expected duration <60 min; surgery involving the arms; need for rapid sequence intubation; surgery that required absolute immobility or prone position; and conditions requiring postoperative mechanical ventilation.

### Anesthesia

All patients arrived in the operating room with a peripheral intravenous (IV) line. Standard monitors were applied with oxygen saturation measured on the IV limb and non-invasive blood pressure on the contralateral limb. Anesthesia was induced with administration of 5 mg·kg^−1^ of thiopental (aged <3 years) or 0.5 mg kg^−1^ of 1% lidocaine, followed by 2–2.5 mg kg^−1^ of propofol (aged ≥3 years). After loss of consciousness, calibration was performed followed by administration of 0.6 mg kg^−1^ of rocuronium. Patients were manually ventilated with sevoflurane in 100% oxygen at 6 L min^−1^ of fresh gas flow. After confirmation of full relaxation by neuromuscular monitoring, endotracheal intubation was performed. During surgery, anesthesia was maintained at 1–1.5 minimum alveolar concentration of sevoflurane. Remifentanil was administered at 0.1–0.2 μg kg^−1^ min^−1^. Additional boluses of rocuronium were administered as required. During anesthesia, blood pressure and heart rate were maintained according to the individual ward measurement. Ventilation was adjusted to a tidal volume of 7 ml^−1^ kg^−1^, and the respiratory rate was adjusted to maintain E_T_CO_2_ of 35–40 mmHg. Intraoperative hypotension was managed with additional fluid bolus, 5–10 mg kg^−1^ of calcium gluconate, 0.05–0.1 mg kg^−1^ of ephedrine, or continuous infusion of dopamine (5 mcg kg^−1^ min^−1^) as clinically indicated. Hypertension was managed by increasing the concentration of inhalation anesthesia or the infusion rate of remifentanil after determining the etiology. Temperature was monitored using either an esophageal or axillary temperature probe. Temperature was maintained between 35.6 and 37.5°C using an over-body forced-air warmer and under-body warming mattress.

During anesthetic recovery, TOF ratio measurements were obtained. After clinical evaluation suggesting adequate airway patency (Davis, 2017) and TOF ratio reaching 0.9, tracheal extubation was performed by an anesthesiologist with >1 year of experience in pediatric anesthesia.

### Monitoring of neuromuscular blockade

**Fig. 1:**
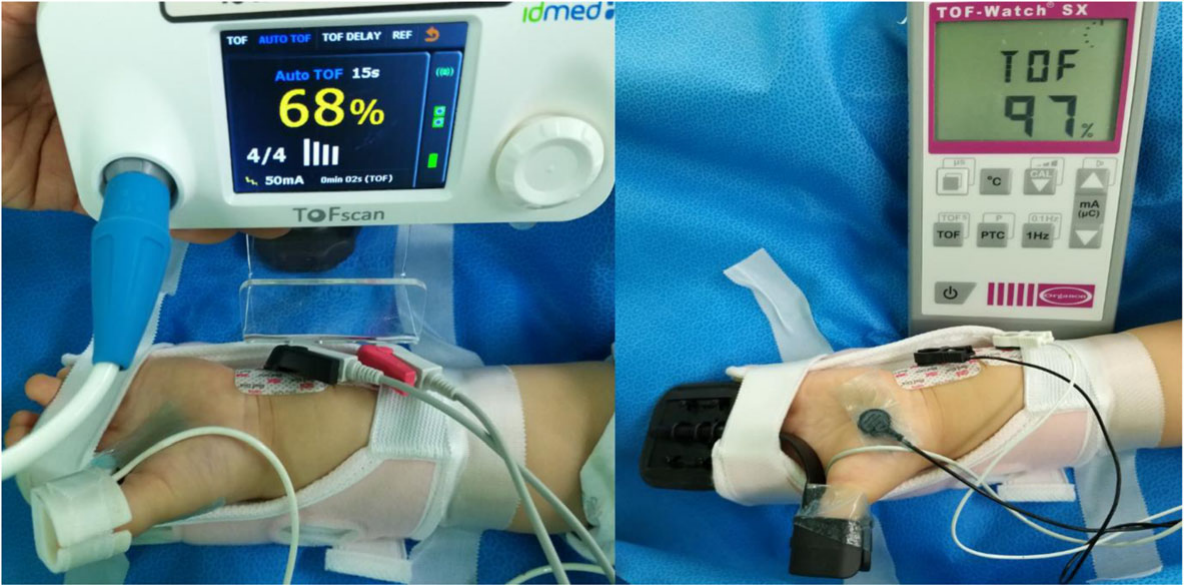
Placement of electrodes on the ulnar nerve and other adapters to the thumb with the other four fingers fixed with adhesive tape; TOFscan (left) and TOF-Watch SX (right)

After loss of consciousness, neuromuscular monitoring was simultaneously initiated using TOF-Watch SX in one forearm and TOFscan in the opposite, with the patient in the supine position. Upper forearms with supinated palm were passively extended and fixed to an arm board to ensure sole movement of adductor pollicis brevis (APB) (Fig. 1). After abrasion and cleansing with an alcohol swab, the skin surface was allowed to dry. Two surface electrodes were placed along the course of the ulnar nerve, with the negative electrode at a distal location near the styloid process of the radius and the positive electrode at 3 cm proximally. An adult or a pediatric hand sensor of TOFscan was used according to the size of the patient’s hand. TOF-Watch SX was calibrated using the CAL2 function (Martin-Flores et al., 2012). Time to acquire the baseline TOF-Watch SX calibration was measured. TOFscan was initiated with non-calibrated intensity of stimulation fixed at 50 mA for all patients (Colegrave et al., 2016). TOF stimuli from the two devices were repeated every 15 s until after surgery. Prior to extubation, neuromuscular blockade antagonism was made with atropine 15 mcg kg^−1^ and neostigmine 30 mcg kg^−1^ if reversal administration was indicated. TOF ratio ≥0.9 was considered complete neuromuscular function recovery for safe extubation.

### Data analysis

Data were collected using the manufacturer-provided software. The time to reach TOF count 0 was measured. Then, the baseline TOF ratio in TOF-Watch SX’s deviation from 1.0 was adjusted for normalization (Murphy et al., 2018). Likewise, if the baseline TOF ratio with TOF-Watch SX after calibration was 0.9, all block levels (0.1, 0.2, 0.3, 0.4, 0.5, 0.6, 0.7, 0.8, 0.9, and 1.0) were divided by 0.9, yielding the following TOF-Watch values representative of TOF ratio: 0.11, 0.22, 0.33, 0.44, 0.56, 0.67, 0.78, 0.89, 1.00, and 1.11. The TOFscan’s TOF ratio was recorded at the time when the representative values of TOF-Watch SX value were reached. However, non-normalization values do not take into account baseline TOF ratio. Non-normalization only detects matching absolute values of TOF ratio between the two devices at levels of TOF ratios 0.1, 0.2, 0.3, 0.4, 0.5, 0.6, 0.7, 0.8, 0.9, and 1.0. Additionally, intraoperative NMBA use data, including the initial and additional doses, was collected. If additional doses of NMBAs were administered, the TOF ratio was collected again, and the same process was repeated.

The primary endpoint was the measure of TOFscan value at TOF-Watch SX’s ratio of 0.7. The final analysis determined bias with limits of agreement (95% confidence interval, CI) for both normalized and non-normalized pairs throughout the TOF ratio of 0.1–1.0. Secondary outcomes included results of the expanded Bland-Altman method, referred to as modified true value varies analysis (Olofsen et al., 2014).

### Sample size estimation

The sample size to assess the agreement between TOF-Watch SX and TOFscan was calculated according to a previously established methodology (Murphy et al., 2018). The results were bias of 0.021 (standard deviation [SD] = 0.009) for non-normalized and 0.015 (SD=0.008) for normalized values. With these results, we assumed the reliable mean of bias as 0.02 (SD=0.01) regardless of the presence or absence of normalization. We predefined the maximum allowed bias (δ) as 0.05, while setting the minimum power as 0.90. Overall, 35 pediatric patients were required; assuming a dropout rate of 10%, we planned to enroll 39 participants.

### Statistical analysis

Bland-Altman analysis was used to evaluate the agreement over repeated measurements at each block level. Limits of agreement were calculated as the means of differences between two measurements ± 1.96×SD, resulting in 95% CI upper and lower limits. Furthermore, the bias was tested for normal distribution by Shapiro-Wilk test, along with probability quantile-quantile plot. To evaluate homogeneity of variances across the measurement range, correlation between the size of the bias and the mean values was measured through Spearman rank correlation and Kendall rank correlation coefficient.

Statistical analyses were performed using R programming version 3.6.1 and blandr (version 0.5.1) package and SPSS version 25.0 (IBM Corp., Armonk, NY). Continuous variables, such as patient characteristics and intraoperative measurements, were analyzed using Student’s *t*-test. Categorical variables were analyzed using Pearson’s chi-squared test. Values are expressed as mean ± SD with corresponding 95% CI, or median (interquartile rage). Normality of the distribution was determined using the Shapiro-Wilk test. A *P*-value <0.05 was considered statistically significant.

## Results

In total, 39 children were screened, of whom 35 were enrolled. Demographic characteristics are shown in Table 1. Four patients were excluded due to missing data, and 16 required additional NMBA maintenance. Although the supramaximal current was secured, the TOF-Watch SX ratio of 12 children ended before reaching 1.0 due to short surgical time. Therefore, 12 data pairs of TOF ratio 1.0 in both normalized and non-normalized groups were missing. However, all 35 data pairs were secured until a TOF ratio of 0.9.

**Table 1:** Demographics of study population

Characteristics	*N* = 35
Age	9 [8–11]
Height (cm)	140.0 [131.0–151.5]
Weight (kg)	38.0 [28.0–46.5]
M/F	14/21
Surgery time (min)	125.0 [90.0–175.0]
Anesthesia time (min)	160.0 [117.5–217.5]

Values are numbers or median [IQR, 25–75%]

Baseline TOF ratio was 0.029 higher (*P*=0.026) for TOF-Watch SX than TOFscan (1.026±0.073 and 0.997±0.009, respectively). The time to reach TOF count 0 was shorter in TOF-Watch SX (100.5±34.6 s) than in TOFscan (112.1±34.6 s, *P*=0.018). Average calibration time for TOF-Watch SX was 36.88 s. At a TOF ratio of 0.7 in TOF-Watch SX, the TOF ratio of TOFscan was similar (0.712±0.116, *P*=0.545; Table 2).

**Table 2:** Baseline TOF ratio with the onset of NMBA between TOF-Watch SX and TOFscan

Data	TOF-Watch SX	TOFscan	Difference (95% CI)	*P*-value
Baseline TOF ratio	1.026±0.073	0.997±0.009	0.029 (0.004, 0.054)	0.026
Calibration time (s)	38.68±17.08			
Time to TOF count 0 (s)	100.5±34.6	112.1±34.6	11.5 (−7.7, 30.8)	0.018
TOFscan value at TOF-Watch 0.7	0.700±0.000	0.712±0.116	−0.012 (−0.052, 0.028)	0.545

Data are reported as mean ± SD and were compared using the paired *t* test. *n* = 35

**Fig. 2:**
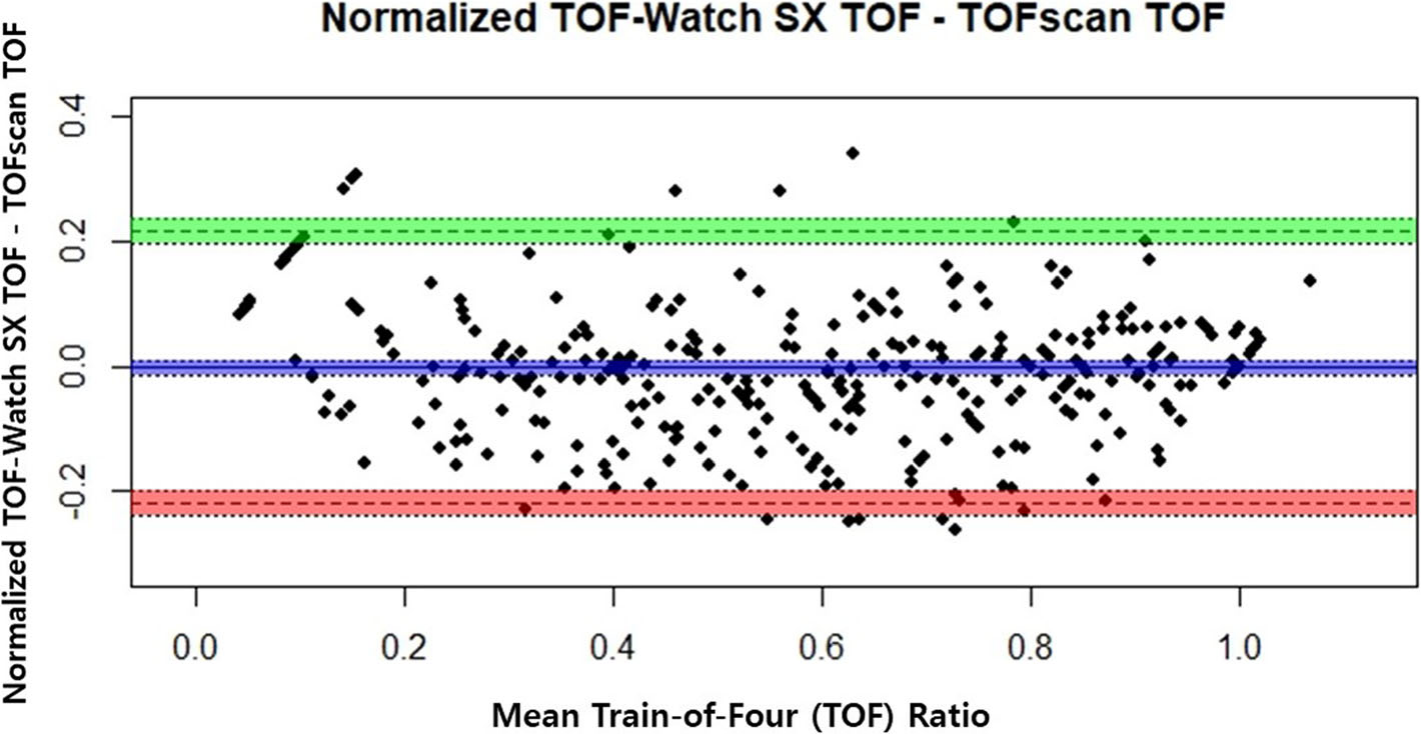
Normalized Bland-Altman plot for the difference in the train-of-four ratio values between TOF-Watch SX and TOFscan the during recovery phase. Bias and its 95% CI (*blue*), 95% upper limit of agreement and its 95% CI (*green*), and 95% lower limit of agreement and its 95% CI (*red*) are illustrated

In non-normalized TOF ratios, the bias was 0.010 (95% CI, −0.003 to 0.0236) with SD of 0.127. The 95% limits of agreement were −0.239 to 0.259 for non-normalized ratios. The CI for lower limit of agreement was −0.263 to −0.216, and CI for the upper limit of agreement was 0.235 to 0.282. Normalized TOF-Watch SX and TOFscan data showed bias of −0.002 (95% CI, −0.013 to 0.010) with SD of 0.111. The 95% limits of agreement were −0.219 to 0.216 for normalized ratios. The CI for lower limit of agreement was −0.239 to −0.119, and CI for the upper limit of agreement was 0.196 to 0.236 (Table 3). A discrepancy was detected in seven datasets because of additional TOF ratio gained at TOF ratio of 1.0 in the normalization process (non-normalized, 338, vs normalized, 345). Each measurement is displayed in the Bland-Altman plot along with each 95% CI shown in green (upper 95%) and red (lower 95%) (Fig. 2, Supplement 1).

**Table 3:** Bias and 95% limits of agreement in non-normalized and normalized TOF-Watch SX and TOFscan

TOF-Watch SX measurements	Bias ± standard error (TOF-Watch SX – TOFscan; 95% CI)	SD ± standard error of the differences	95% Limits of agreement	95% CI, lower limit of agreement	95% CI, upper limit of agreement
Non-normalized^a^	0.010±0.007 (−0.003, 0.0236)	0.127±0.0120	−0.239 to 0.259	−0.263 to −0.216	0.235 to 0.282
Normalized^b^	−0.002±0.006 (−0.013, 0.010)	0.111±0.010	−0.219 to 0.216	−0.239 to −0.199	0.196 to 0.236

^a^338 measurements in 35 individuals

^b^345 measurements in 35 individuals

The Spearman rank correlation coefficients (ρ) were −0.237 and −0.236 for non-normalized and normalized biases, respectively. Similarly, Kendall rank correlation coefficients (*τ*) were −0.164 and −0.160 for non-normalized and normalized groups, respectively. This suggests a weak relationship in the bias over the range of measurements, regardless of normalization. The intraclass correlation coefficients were 0.921 and 0.929 for non-normalized and normalized biases, respectively, indicating the high reliability of both devices, regardless of normalization (Table 4).

**Table 4:** Correlation coefficient and intraclass correlation in non-normalized and normalized TOF-Watch SX and TOFscan

TOF-Watch SX Measurements	Spearman rank correlation ^a^ coefficient (*ρ*)	Kendall rank correlation ^a^ coefficient (*τ*)	Intraclass correlation^c^ (95% CI)
Non-normalized	−0.237^b^	−0.164^b^	0.921 (0.863 to 0.962)
Normalized	−0.236^b^	−0.160^b^	0.929 (0.914 to 0.943)

^a^The correlation between the size of the bias and the mean values of corresponding TOF ratio

^b^Correlation is significant at the 0.01 level (2-tailed)

^c^The ratio of the between-subject variance and the total variance

## Discussion

This study presents lack of bias and good concordance between TOF-Watch SX and TOFscan in children. The normalized group displayed less absolute value of bias with smaller SD and narrower bidirectional (upper and lower) limits of agreement than the non-normalized group. Normalized values coincided with those of TOFscan more accurately than the non-normalized values. The Spearman rank correlation coefficient (*ρ*) and Kendall rank correlation coefficient (*τ*) of normalized and non-normalized measurements indicated a weak correlation over the course of neuromuscular recovery.

Three-dimensional acceleromyography devices, such as TOFscan, Stimpod NMS 450 (Xavant, South Africa), and Mindray neuromuscular transmission transducer (Shenzhen, China), use three perpendicular piezoelectric probes to thoroughly measure freely moving target muscles. To date, only two studies, both in adults, have compared the performances of TOF-Watch SX and TOFscan. One reported no significant differences between the two but suggested that TOF-Watch SX is more sensitive during deep neuromuscular blockade and that better agreement was observed along the recovery (Colegrave et al., 2016). Another study detected minimal bias between these devices (Murphy et al., 2018). Our findings also indicate minimal bias; the absolute value of bias was also smaller. Also, Spearman rank and Kendall rank correlation analysis revealed a weak negative relationship. It indicates that size of bias did not significantly change but that a decreasing trend over bias was observed during neuromuscular recovery. This result supports previous observations of a similar but slight negative trend in adults (Murphy et al., 2018) and observations that better agreement was achieved at more complete levels of recovery (Colegrave et al., 2016).

Assessing three-dimensional acceleration in children is challenging due to difficulty in isolating APB movement. The APB originates from two heads (oblique head from the second and third metacarpal bones, and transverse head from the third metacarpal bone) and inserts at the base of the thumb’s proximal phalanx. Contraction of the APB brings the thumb’s tip to the center of the palm. However, adjacent muscles, such as the opponens pollicis (OP) and flexor pollicis brevis (FPB) muscles, also contribute to similar movements. Given the shallow skin-to-nerve and nerve-to-nerve distance in pediatric patients, applying same the “3-cm” distance between the positive and negative electrodes can cause extra stimulation to the median nerve, which innervates OP and FPB (Alanazy, 2017). The distance between the two electrodes determines the penetration depth (Fuchs-Buder, 2011). With the distance between the electrodes relatively far apart in the relatively short pediatric forearm, nearby muscles may also react. Strapping prevents only the four fingers, not the thumb from moving. OP and FPB cannot be restricted by mere strapping. Moreover, the deep part of the OP and FPB in 20% of the population is often innervated by the deep branch of the ulnar nerve (C8, T1) (Gupta & Michelsen-Jost, 2012). The erroneous extra-apposition of the thumb due to the mixed effect of unwanted muscle movement is likely to occur in a pediatric patient with smaller hands and more proximal nerve-to-nerve distance. This anatomical inevitability may cause unwanted acceleration and induce a larger correlation coefficient than that demonstrated in Murphy et al.’s previous report.

Normalization of the TOF-Watch values resulted in lower bias, narrower limits of agreement, and higher intraclass correlation between devices. TOF ratio >1 has been previously demonstrated in TOF-Watch SX (Suzuki et al., 2006; Claudius & Viby-Mogensen, 2008; Bowdle et al., 2019). Without normalization, if the initial TOF exceeds 1, subsequent TOF ratios will be overestimated. Alternatively, if initial TOF <1, subsequent ratios may be underestimated. Individual differences in distribution volume, muscle mass, NMBA clearance, and age-dependent maturation of neuromuscular junction may contribute to differences in initial TOF ratio. As normalization adjusts the inherent TOF ratio, the results support the use of normalized TOF ratios to provide a more accurate TOF ratio along the recovery in children (Suzuki et al., 2006; Claudius et al., 2009).

The completion of TOF count 0 in TOFscan was longer by approximately 12 s. According to previous studies, the time to TOF count 0 showed inconsistent results between TOF devices (Colegrave et al., 2016; Murphy et al., 2018). The reason for the discrepancy may be associated with the calibration process. The maximal value, the current that gives 100% response, is acquired with a calibration mode called CAL2, and 110% of maximal value is suggested as the supramaximal reference (Martin-Flores et al., 2012; Schreiber et al., 2011). Compared with TOFscan, which delivers uniform current intensity of 50 mA at 2 Hz, the calibration process in TOF-Watch provides 10 stimuli at 1 Hz until the optimal supramaximal current is detected. Such higher initial frequency may affect the gradual potentiation process—in other words, a staircase phenomenon (Zhou et al., 2013). The staircase phenomenon influences the onset time and duration of twitch depression by increasing T1 values (Martin-Flores et al., 2011). Nonetheless, some researchers argue that, despite the staircase phenomenon, T1 through T4 increase in the same proportion, therefore not affecting the TOF ratio (Suzuki et al., 2006). However, others reported the need for an extra-stabilization period after the staircase phenomenon to obtain a stable baseline (Martin-Flores et al., 2011). Therefore, calibration process may either effect T1 size or time to reach a stable T1 to T4 baseline. As such, further investigation is required to determine the reliability of the measurement devices at TOF count 0.

This study has some limitations. First, we did not compare acceleromyography with mechanomyography, despite acceleromyography itself not differing significantly from mechanomyography, especially when calibrated and normalized (Bowdle et al., 2019; Claudius et al., 2010). Second, no randomization was performed between the right and left arm, but previous research indicates that arm-to-arm variations did not display significant bias gap (Claudius et al., 2010; Hohenauer et al., 2017). Third, preload and device size were not individualized. However, acceleromyography is well reported to be precise with preload application ranging from 75–120 g (Claudius et al., 2009). Although we used a pediatric sensor, the TOFscan’s sensor might be too big for very small children. To correctly measure acceleration, the index finger should fit in the sensor’s hole after the curvature. Our youngest patient was a 16-month-old child whose finger length fit the sensor. However, to expand TOFscan’s validity to infants and neonates, a smaller sensor is required. Fourth, we did not identify the potential effect of calcium in neuromuscular monitoring. In motor neurons, presynaptic voltage-gated calcium channel activation produces neuromuscular junction’s synaptic vesicle to release acetylcholine, which eventually causes muscle contraction. Some studies report that increased ionized calcium levels decrease sensitivity to non-depolarizing NMBA and enhance neuromuscular recovery (Ju et al., 2017; Munir et al., 2003). When comparing TOF ratio, equalizing the use of calcium between groups would be helpful. Finally, our results reliably indicated concordance and lack of bias but no agreement between the devices. The limits of agreement’s absolute range exceeded 0.2, and this difference in TOF ratio indicates poor agreement. Careful interpretation is required since a 0.2 difference practically results in diverse decisions regarding extubation time and reversal dose. In conclusion, TOFscan demonstrated good concordance and is unbiased with TOF-Watch SX in children’s neuromuscular recovery, especially when normalized.

## Supplementary Information

**Additional file 1: Supplement 1**. Non-normalized Bland-Altman plot for the difference in the train-of-four ratio values between TOF-Watch SX and TOFscan during the recovery phase. Bias and its 95% CI (*blue*), 95% upper limit of agreement and its 95% CI (*green*), and 95% lower limit of agreement and its 95% CI (*red*) are illustrated.

**Additional file 2: Supplement 2**. Probability quantile-quantile plot (Q-Q plot) for the differences in normalized group. Mean and SD of data are -0.0065 and 0.13. Shapiro-Wilk test statistics = 0.992 (*P* = 0.078), indicating that differences are normally distributed.

## Data Availability

The datasets used and/or analyzed during the current study are available from the corresponding author on reasonable request.

## Abbreviations

NMBA: Neuromuscular blocking agent
IV: Intravenous cannulation
APB: Adductor pollicis brevis
OP: Opponens pollicis
FPB: Flexor pollicis brevis
CI: Confidence interval
WFSA: World Federation of Societies of Anaesthesiologists
SFAR: French Society for Anesthesia and Intensive Care

## References

[CR1] Alanazy M (2017). Clinical and electrophysiological evaluation of carpal tunnel syndrome: approach and pitfalls. Neurosciences..

[CR2] Bowdle A, Bussey L, Michaelsen K, Jelacic S, Nair B, Togashi K, Hulvershorn J (2019). A comparison of a prototype electromyograph vs. a mechanomyograph and an acceleromyograph for assessment of neuromuscular blockade. Anaesthesia..

[CR3] Brull SJ, Kopman AF (2017). Current status of neuromuscular reversal and monitoring. Anesthesiology..

[CR4] Claudius C, Skovgaard LT, Viby-Mogensen J (2009). Is the performance of acceleromyography improved with preload and normalization?. Anesthesiology..

[CR5] Claudius C, Skovgaard LT, Viby-Mogensen J (2010). Arm-to-arm variation when evaluating neuromuscular block: an analysis of the precision and the bias and agreement between arms when using mechanomyography or acceleromyography. Br J Anaesth.

[CR6] Claudius C, Viby-Mogensen J (2008). Acceleromyography for use in scientific and clinical practice. Anesthesiology..

[CR7] Colegrave N, Billard V, Motamed C, Bourgain J-L (2016). Comparison of the TOF-Scan™ acceleromyograph to TOF-Watch SX™: Influence of calibration. Anaesth Critical Care Pain Med.

[CR8] Davis PJCF (2017). Smith’s anesthesia for infants and children: Elsevie.

[CR9] de Souza CM, Romero FE, Tardelli MA (2011). Assessment of neuromuscular blockade in children at the time of block reversal and the removal of the endotracheal tube. Braz J Anesthesiol.

[CR10] Fortier L-P, McKeen D, Turner K, de Médicis É, Warriner B, Jones PM, Chaput A, Pouliot JF, Galarneau A (2015). The RECITE Study. Anesth Analg.

[CR11] Fuchs-Buder T (2011). Neuromuscular monitoring: Springer Science & Business Media.

[CR12] Gelb AW, Morriss WW, Johnson W, Merry AF, Abayadeera A, Belîi N, Brull SJ, Chibana A, Evans F, Goddia C, Haylock-Loor C, Khan F, Leal S, Lin N, Merchant R, Newton MW, Rowles JS, Sanusi A, Wilson I, Velazquez Berumen A, International Standards for a Safe Practice of Anesthesia Workgroup (2018). World Health Organization-World Federation of Societies of Anaesthesiologists (WHO-WFSA) International Standards for a Safe Practice of Anesthesia. Anesth Analg.

[CR13] Gupta S, Michelsen-Jost H (2012). Anatomy and Function of the Thenar Muscles. Hand Clin.

[CR14] Hardman JG, Wills JS (2006). The development of hypoxaemia during apnoea in children: a computational modelling investigation. Br J Anaesth.

[CR15] Hohenauer E, Cescon C, Deliens T, Clarys P, Clijsen R (2017). The effect of local skin cooling before a sustained, submaximal isometric contraction on fatigue and isometric quadriceps femoris performance: A randomized controlled trial. J Thermal Biol.

[CR16] Ju J-W, Kim H-C, Yoon S, Hong DM, Park H-P (2017). Effects of calcium chloride coadministered with neostigmine on neuromuscular blockade recovery. Eur J Anaesthesiol.

[CR17] Ledowski T, O’Dea B, Meyerkort L, Hegarty M, von Ungern-Sternberg BS (2015). Postoperative residual neuromuscular paralysis at an Australian tertiary children’s hospital. Anesthesiol Res Pract.

[CR18] Martin-Flores M, Gleed RD, Basher KL, Scarlett JM, Campoy L, Kopman AF (2012). TOF-Watch ® monitor: failure to calculate the train-of-four ratio in the absence of baseline calibration in anaesthetized dogs. Br J Anaesth.

[CR19] Martin-Flores M, Lau EJ, Campoy L, Erb HN, Gleed RD (2011). Twitch potentiation: a potential source of error during neuromuscular monitoring with acceleromyography in anesthetized dogs. Vet Anaesth Analg.

[CR20] Munir MA, Jaffar M, Arshad M, Akhter MS, Zhang J (2003). Reduced duration of muscle relaxation with rocuronium in a normocalcemic hyperparathyroid patient. Can J Anaesth.

[CR21] Murphy GS, Szokol JW, Avram MJ, Greenberg SB, Shear T, Vender JS, Gray J, Landry E (2013). Postoperative residual neuromuscular blockade is associated with impaired clinical recovery. Anesth Analg.

[CR22] Murphy GS, Szokol JW, Avram MJ, Greenberg SB, Shear TD, Deshur M, Benson J, Newmark RL, Maher CE (2018). Comparison of the TOFscan and the TOF-Watch SX during recovery of neuromuscular function. Anesthesiology..

[CR23] Naguib M, Brull SJ, Johnson KB (2017). Conceptual and technical insights into the basis of neuromuscular monitoring. Anaesthesia..

[CR24] Naguib M, Brull SJ, Kopman AF, Hunter JM, Fülesdi B, Arkes HR, Elstein A, Todd MM, Johnson KB (2018). Consensus statement on perioperative use of neuromuscular monitoring. Anesth Analg.

[CR25] Nemes R, Renew JR (2020). Clinical practice guideline for the management of neuromuscular blockade: what are the recommendations in the USA and other countries?. Curr Anesthesiol Rep.

[CR26] Olofsen E, Dahan A, Borsboom G, Drummond G (2014). Improvements in the application and reporting of advanced Bland–Altman methods of comparison. J Clin Monit Comput.

[CR27] Plaud B, Baillard C, Bourgain J-L, Bouroche G, Desplanque L, Devys J-M, Fletcher D, Fuchs-Buder T, Lebuffe G, Meistelman C, Motamed C, Raft J, Servin F, Sirieix D, Slim K, Velly L, Verdonk F, Debaene B (2020). Guidelines on muscle relaxants and reversal in anaesthesia. Anaesth Critical Care Pain Med.

[CR28] Schreiber JU, Mucha E, Fuchs-Buder T (2011). Acceleromyography to assess neuromuscular recovery: is calibration before measurement mandatory?. Acta Anaesthesiol Scand.

[CR29] Suzuki T, Fukano N, Kitajima O, Saeki S, Ogawa S (2006). Normalization of acceleromyographic train-of-four ratio by baseline value for detecting residual neuromuscular block. Br J Anaesth.

[CR30] von Ungern-Sternberg BS, Hammer J, Schibler A, Frei Franz J, Erb TO (2006). Decrease of functional residual capacity and ventilation homogeneity after meeting abstracts in anesthetized young infants and preschool children. Anesthesiology..

[CR31] Zhou Z-J, Wang X, Zheng S, Zhang X-F, Lerman J (2013). The characteristics of the staircase phenomenon during the period of twitch stabilization in infants in TOF mode. Pediatr Anesth.

