# Supplementary figures and images for: Comparison of the TOFscan and the TOF-Watch SX during pediatric neuromuscular function recovery: a prospective observational study
Source: Perioper Med (Lond). 2021 Dec 10;10:45. doi: 10.1186/s13741-021-00215-2 (PMC8662865; doi:10.1186/s13741-021-00215-2)

Supplement 1. Non-normalized Bland-Altman plot

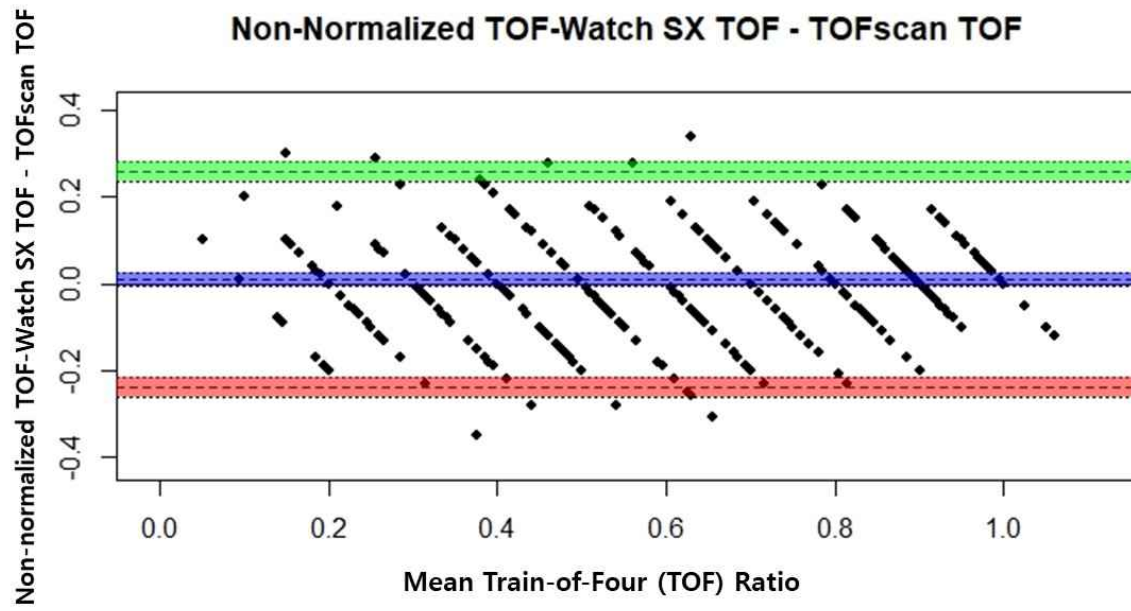

Supplement: Supplementary file 1 — Additional file 1: Supplement 1. Non-normalized Bland-Altman plot for the difference in the train-of-four ratio values between TOF-Watch SX and TOFscan during the recovery phase. Bias and its 95% CI (blue), 95% upper limit of agreement and its 95% CI (green), and 95% lower limit of agreement and its 95% CI (red) are illustrated. [file 13741_2021_215_MOESM1_ESM.pdf]

Supplement 2. Probability plot for the differences.

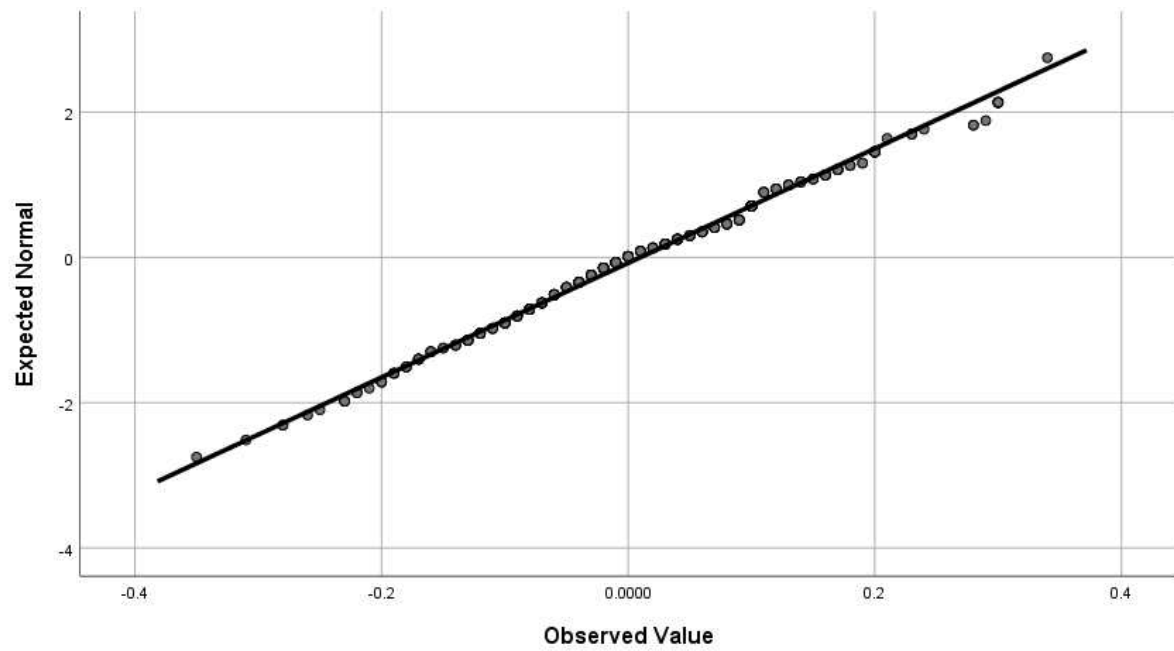

Supplement: Supplementary file 2 — Additional file 2: Supplement 2. Probability quantile-quantile plot (Q-Q plot) for the differences in normalized group. Mean and SD of data are -0.0065 and 0.13. Shapiro-Wilk test statistics = 0.992 (P = 0.078), indicating that differences are normally distributed. [file 13741_2021_215_MOESM2_ESM.pdf]
